# Integrated transcriptomic profiling of rectal and adipose HIV-1 reservoirs relative to matched PBMCs in ART-treated individuals

**DOI:** 10.3389/fimmu.2026.1873668

**Published:** 2026-07-07

**Authors:** Jie Qin, Peiming Huang, Minghua Chen, Hua Zong, Jingjing Luo, Jianteng Zeng, Xu Zhang, Shiting Huang, Jianqiang Zou, Xin He, Shuangxin Wu, Liqin Sun, Ting Pan

**Affiliations:** 1 Department of Gastrointestinal Surgery, Shenzhen Third People’s Hospital, The Second Hospital Affiliated to Southern University of Science and Technology, Shenzhen, Guangdong, China; 2 Shenzhen Key Laboratory for Systems Medicine in Inflammatory Diseases, Zhongshan School of Medicine, Sun Yat-Sen University, Shenzhen, Guangdong, China; 3 Institute of Human Virology, Department of Pathogen Biology and Biosecurity, Key Laboratory of Tropical Disease Control of Ministry of Education, Zhongshan School of Medicine, Sun Yat-sen University, Guangzhou, Guangdong, China; 4 Medical Research Center, the Eighth Affiliated Hospital of Sun Yat-sen University, Shenzhen, Guangdong, China; 5 Department Infectious Diseases, National Clinical Research Center for Infectious Diseases, Shenzhen Third People’s Hospital, The Second Hospital Affiliated to Southern University of Science and Technology, Shenzhen, Guangdong, China

**Keywords:** anatomical sanctuary, HIV-1 latent reservoirs, PI3K-Akt signaling pathway, tissue remodeling, transcriptomic profiling

## Abstract

**Background:**

HIV-1 persistence in anatomical reservoirs remains the primary obstacle to a functional cure, even under suppressive antiretroviral therapy (ART). While gut-associated lymphoid tissue (GALT) is a well-recognized site of viral sequestration, the role of adipose tissue as an immunometabolic sanctuary, and its distinct host immune environment compared with peripheral blood remain under-characterized.

**Methods:**

In this study, we quantified the HIV-1 DNA reservoir using droplet digital PCR (ddPCR) in rectal tissues and subcutaneous adipose tissues (SAT), each paired with matched peripheral blood mononuclear cells (PBMCs) from ART-treated individuals. To characterize the host transcriptomic landscape associated with viral persistence, RNA-seq was performed across these compartments. An external healthy human tissue dataset (GSE120795) was integrated to perform background-identity filtering. Key differentially expressed signatures and enriched pathways were further validated using RT-qPCR and Western blot.

**Results:**

The ddPCR analysis confirmed significantly higher viral DNA loads in both rectal and adipose tissues relative to matched PBMCs, identifying them as major anatomical reservoirs. Transcriptomic profiling after background filtering revealed divergent host responses: rectal reservoirs were primarily characterized by extracellular matrix (ECM) reorganization and epithelial barrier dysfunction, whereas SAT exhibited prominent dysregulation in cell cycle progression and immunometabolic signaling. Notably, cross-tissue enrichment analysis identified the PI3K-Akt signaling pathway as a common transcriptomeic feature across these anatomical sites. Validation experiments confirmed that fibrosis-associated hub gene *COL1A1* and the leukocyte recruitment marker *CX3CR1* exhibited tissue-specific expression patterns at both mRNA and protein levels compared with PBMCs.

**Conclusion:**

Our study provides a comprehensive landscape of tissue-specific transcriptomic remodeling in rectal and adipose HIV-1 reservoirs. These findings suggest that persistent viral sequestration is associated with distinct microenvironmental alterations, ranging from fibrotic shifts to metabolic dysregulation, which share common molecular features such as potentially linked to the PI3K-Akt axis. This work underscores the importance of targeting anatomical sanctuaries beyond the peripheral blood in future HIV-1 cure strategies.

## Introduction

The advent of ART has transformed HIV-1 infection into a manageable chronic condition (1, 2). However, ART fails to eradicate the virus due to the persistence of stable viral reservoirs, anatomical and cellular sites where HIV-1 survives in latent or low-level replication states, sequestered from immune surveillance and pharmacological intervention (3–5). While resting memory CD4^+^ T cells in the peripheral blood are the most frequently studied, they represent only a minute fraction of the total body viral burden (6, 7). Consequently, investigating anatomical sanctuaries within deep tissues has become a critical imperative for developing effective shock and kill or block and lock eradication strategies (8, 9).

Among these anatomical compartments, the gastrointestinal (GI) tract is a primary site of viral sequestration and immune exhaustion (10–14). The GALT, particularly in the rectal mucosa, possesses a high density of activated memory CCR5^+^ CD4^+^ T cells, rendering it the primary site for massive viral replication and rapid CD4^+^ T cell depletion during the acute phase of infection (15–18). Persistent viral activity within the GI tract under ART is often associated with chronic mucosal inflammation and intestinal permeability (14, 19). This barrier dysfunction facilitates microbial translocation, driving systemic immune activation, a key driver of non-AIDS comorbidities in people with HIV (PWH) (20, 21).

Parallel to mucosal reservoirs, SAT has emerged as a formidable and unique sanctuary for HIV-1 (22–24). Beyond its role in energy storage, AT is now recognized as a complex immunometabolic hub (25). Several factors contribute to its role as a viral sanctuary: the abundance of susceptible CD4^+^ T cells and macrophages, suboptimal penetration of certain antiretrovirals into lipid-rich environments, and a distinct metabolic milieu that may favor viral latency (22, 23, 26, 27). Despite its potential significance, the host transcriptomic responses within the adipose microenvironment, especially in direct comparison to better-characterized sites like the rectum, remain poorly defined in clinical cohorts (28).

Current understanding of tissue-specific persistence is limited by a reliance on single-tissue studies or animal models, which may not fully capture the systemic heterogeneity of the host response in humans (29). High-resolution analyses that integrate precise viral quantification with global gene expression profiling across matched tissue compartments are notably lacking. It remains unclear whether different anatomical reservoirs share common pathways of immune dysregulation or exhibit divergent, site-specific remodeling. Addressing this gap is essential for identifying targeted molecular signatures that facilitate viral persistence across distinct physiological environments.

In this study, we employed ddPCR to precisely quantify the HIV-1 DNA burden in rectal and adipose tissues relative to matched PBMCs from individuals on long-term ART. By integrating these measurements with transcriptomic profiling (RNA-seq) and adjusting for tissue-baseline signatures using an external healthy control dataset, we mapped the host molecular landscape of these anatomical reservoirs. Our analysis identified shared transcriptional profiles across tissue compartments, highlighted by a common enrichment of pathways related to extracellular matrix remodeling and the PI3K-Akt signaling axis across tissue compartments, which were further characterized through protein-level validation. These findings provide a comprehensive resource for understanding how persistent tissue reservoirs coincide with site-specific and systemic immune remodeling, offering potential molecular markers for future therapeutic intervention.

## Results

### Elevated intact proviral burden in tissues and integrated study design

**Figure 1:**
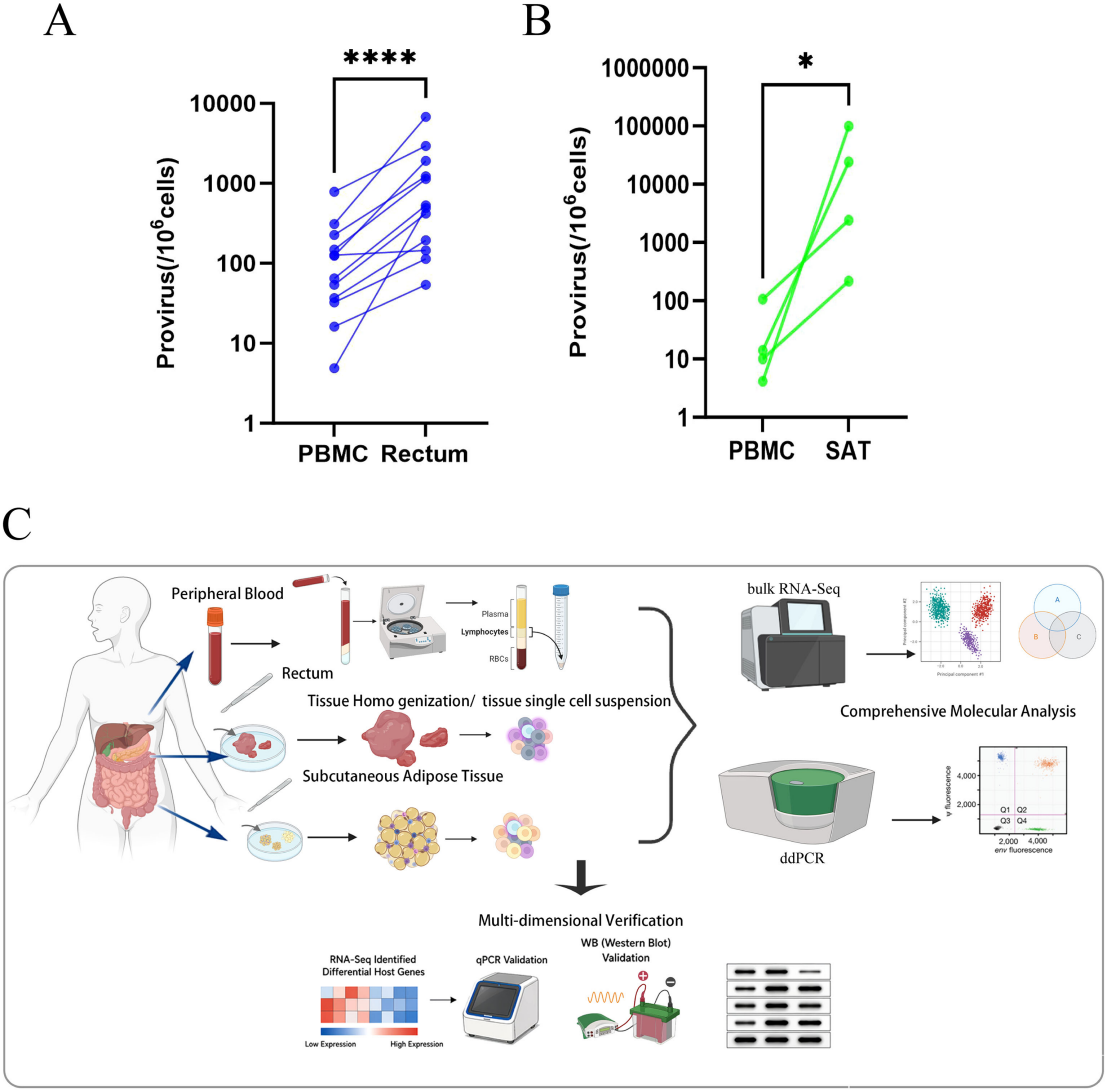
Elevated intact proviral burden in anatomical tissues and study design overview. **(A)** ddPCR quantification of intact proviruses in paired PBMC and rectal tissue samples from PWH (n = 11; copies per 10^6 cells). **(B)** ddPCR quantification of intact proviruses in paired PBMC and SAT samples from PWH (n = 4; copies per 10^6 cells). Each line represents an individual participant. Statistical significance for ddPCR comparisons was determined by paired t-test. **(C)** Schematic overview of the integrated study design, including clinical sample collection, bulk RNA-seq, bioinformatic integration, and experimental validation. *P < 0.05, and ****P < 0.0001.

To characterize the landscape of HIV-1 persistence across anatomical compartments, we first quantified intact proviral DNA levels using ddPCR (30). In paired comparisons, both rectal tissues and SAT exhibited significantly higher intact proviral loads than matched PBMC samples. As shown in the linked-dot plots, intact proviral levels were consistently elevated in the rectum relative to paired PBMCs (**Figure 1A**). A similar trend was observed in SAT, which demonstrated a significant increase in viral sequestration compared with matched PBMCs (**Figure 1B**). These data indicate that these tissue compartments harbor a substantially greater burden of intact viral reservoirs than peripheral blood, reinforcing the role of the rectum and SAT as critical anatomical sanctuaries for HIV-1 persistence during suppressive ART.

Based on these findings, we established an integrated experimental workflow to dissect the host-reservoir interface across different physiological environments (**Figure 1C**). Matched samples (Rectum vs. PBMC and SAT vs. PBMC) were collected from ART-treated individuals, followed by systematic cell isolation for multi-dimensional analysis. High-sensitivity ddPCR was employed for precise viral quantification, while global host transcriptomic features were characterized using bulk RNA-seq. Candidate molecular alterations and core signaling pathways identified via bioinformatic screening were validated at both mRNA and protein levels to ensure robustness. This comprehensive framework provided a robust foundation for identifying both tissue-specific remodeling and shared host responses associated with by persistent viral sequestration.

### Transcriptomic heterogeneity and differential expression across anatomical compartments

**Figure 2:**
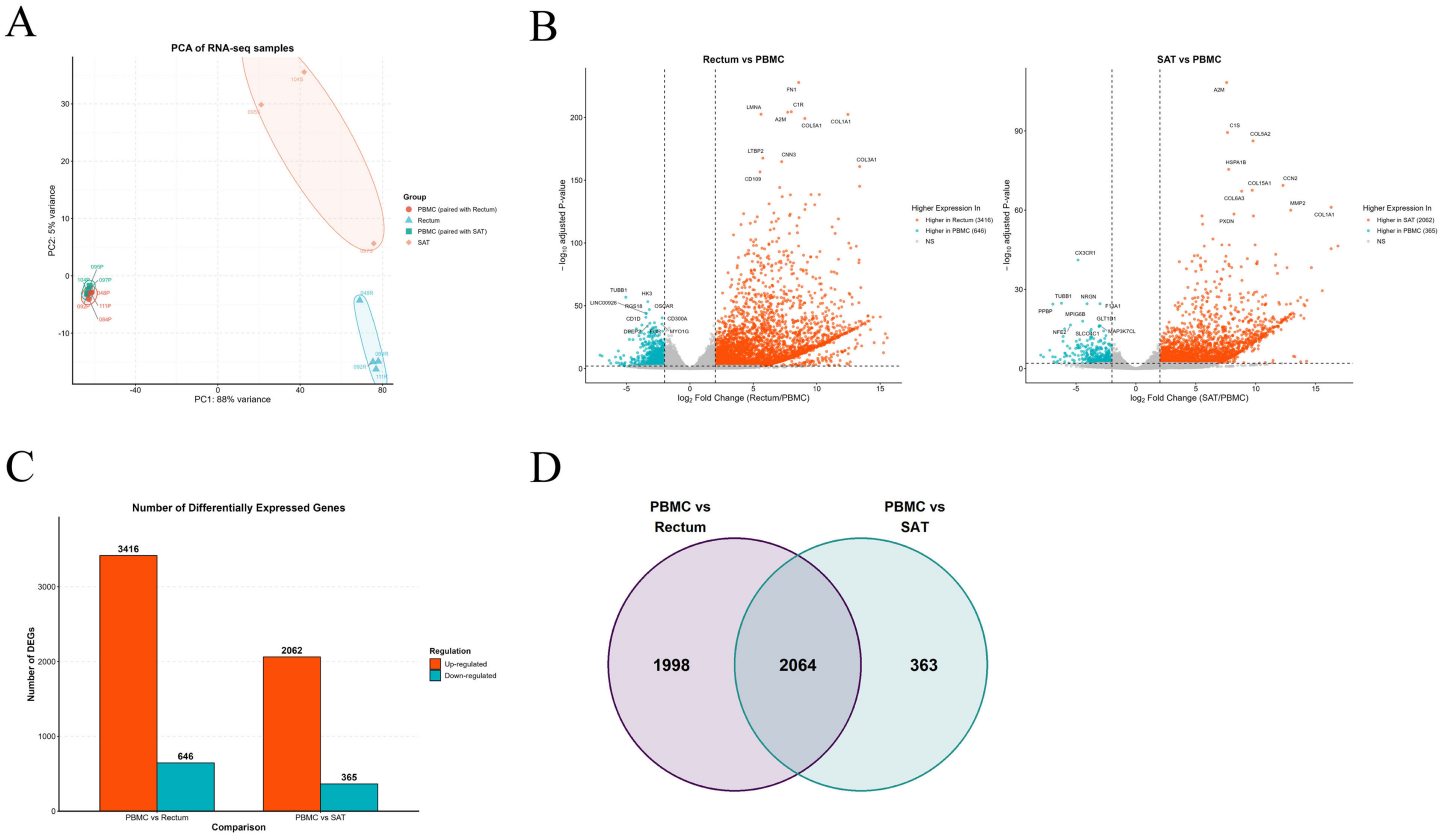
Transcriptomic heterogeneity and differential expression across anatomical compartments. **(A)** PCA of bulk RNA-seq profiles across all sample groups, demonstrating distinct clustering by tissue type. **(B)** Volcano plots highlighting DEGs for Rectum vs PBMC and SAT vs PBMC. Red and blue dots represent significantly up- and down-regulated genes, respectively (adjusted *P* < 0.01 and |log_2_ fold change| > 2). **(C)** Bar plot summarizing the total number of DEGs in both comparisons. Red bars indicate genes upregulated in the corresponding tissue relative to PBMC, whereas blue bars indicate genes downregulated relative to PBMC. **(D)** Venn diagram showing the intersection of DEGs between the rectum and SAT comparisons, identifying tissue-specific and shared transcriptional signatures.

To examine global transcriptomic variation among samples from distinct anatomical sites, we performed principal component analysis (PCA). The PCA revealed a clear separation of samples according to their tissue origin (**Figure 2A**). Principal component 1 (PC1) accounted for 88% of the total variance, while PC2 explained 5%. Notably, PBMC samples clustered closely together, whereas rectum and SAT samples occupied distinct regions and were clearly separated from PBMCs. This pattern indicates marked transcriptomic heterogeneity among the three anatomical compartments and suggests that tissue origin is the primary determinant of global gene expression variation in this cohort.

We next compared the differential expression profiles between tissue reservoirs and peripheral blood. Volcano plots demonstrated that, using predefined thresholds (adjusted *P* < 0.01 and |*log_2_* fold change| > 2), 3,416 genes were upregulated and 646 genes were downregulated in the rectum relative to PBMCs. In the SAT versus PBMC comparison, 2,062 genes were upregulated and 365 genes were downregulated. In both tissue compartments, upregulated genes substantially outnumbered downregulated genes. Furthermore, the greater number of differentially expressed genes (DEGs) in the Rectum vs. PBMC comparison suggests that rectal tissue undergoes more extensive transcriptomic remodeling relative to the peripheral circulation than SAT (**Figures 2B, C**).

To distinguish between shared and tissue-specific transcriptional changes, we performed an overlap analysis of the DEGs. The Venn diagram identified a core set of 2,064 DEGs shared between the Rectum vs. PBMC and SAT vs. PBMC comparisons (**Figure 2D**). Additionally, 1,998 genes were unique to the rectal reservoir, while only 363 genes were specific to SAT. These results indicate that while the rectum and SAT share a subset of transcriptional alterations relative to PBMCs, rectal tissue exhibits a significantly larger repertoire of tissue-specific molecular changes, consistent with a more structurally and immunologically complex local microenvironment in the mucosal sanctuary.

Given that the substantial separation along PC1 largely reflected baseline cellular identities intrinsic to tissue architecture rather than exclusive HIV-induced pathology, we subsequently implemented an external healthy-background filtering strategy to subtract tissue-specific baseline noise and enrich for genuine, reservoir-associated transcriptional signals.

### Healthy-background filtering identifies residual reservoir-associated signatures beyond baseline tissue identity

**Figure 3:**
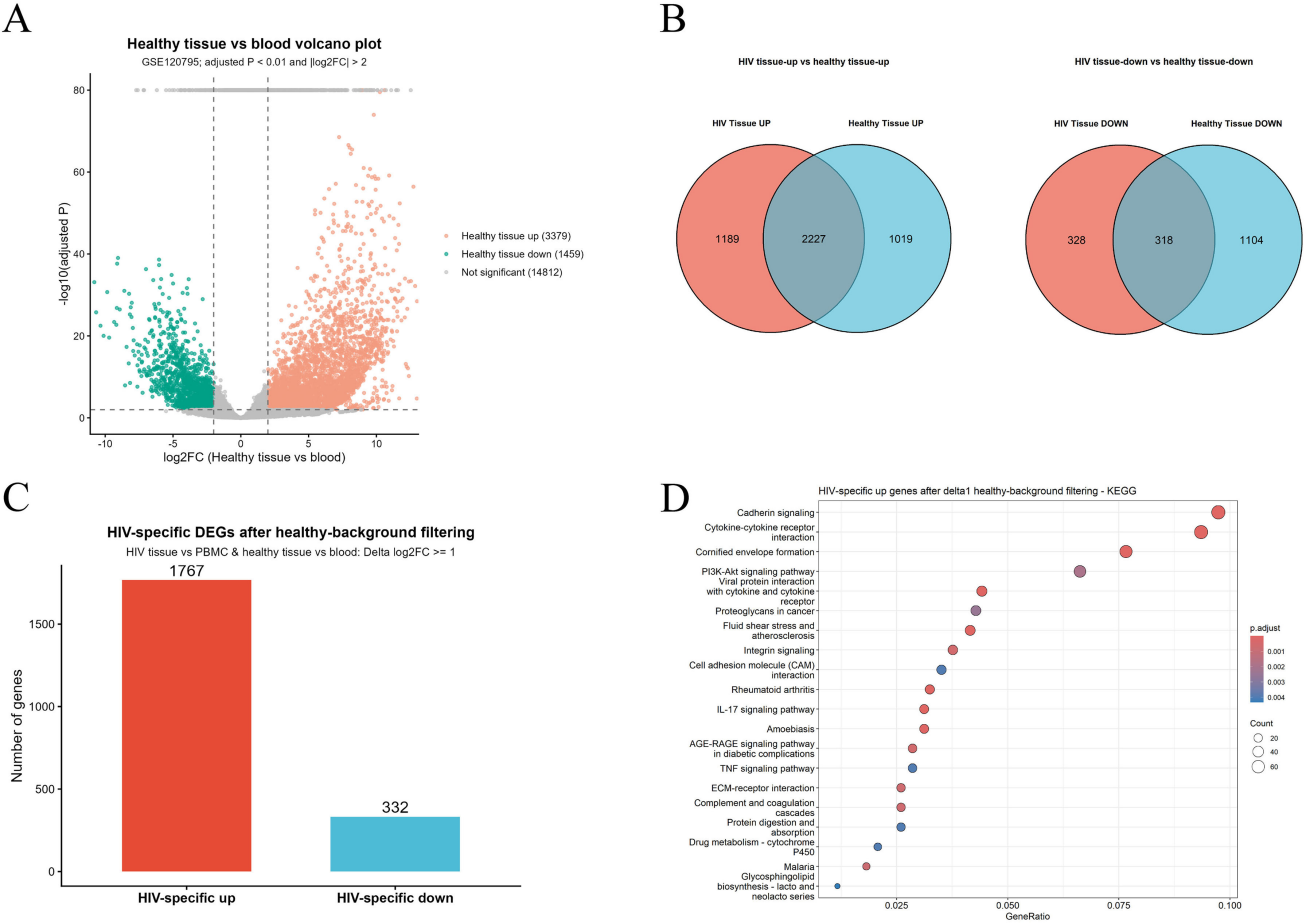
Healthy-background filtering identifies residual transcriptional signatures beyond baseline tissue identity. **(A)** Volcano plot showing DEGs in the healthy tissue-versus-blood comparison using GSE120795. Significant genes were defined as adjusted P < 0.01 and |log2 fold change| > 2. **(B)** Venn diagrams comparing the overlap between HIV tissue-versus-PBMC DEGs and healthy tissue-versus-blood DEGs according to the direction of regulation. **(C)** Bar plot showing the number of healthy-background-filtered putative HIV-associated upregulated and downregulated genes after applying a directional delta log2 fold-change threshold of 1. **(D)** KEGG enrichment analysis of healthy-background-filtered upregulated genes. Dot size represents gene count and color indicates adjusted P value.

Because bulk tissue-versus-PBMC comparisons may be strongly influenced by intrinsic tissue composition, we performed an additional healthy-background filtering analysis using an external normal human tissue transcriptome dataset (GSE120795) (31). This comparison used healthy colon/colon epithelium samples as the tissue reference and whole blood nuclear cell samples as the blood reference. In the healthy tissue-versus-blood comparison, 3,379 genes were upregulated and 1,459 genes were downregulated in healthy tissues relative to blood using the same differential expression thresholds (adjusted P < 0.01 and |log2 fold change| > 2; **Figure 3A**).

We next compared these healthy tissue-background signatures with the DEGs identified in HIV tissue-versus-PBMC comparisons. A substantial fraction of HIV tissue-upregulated genes overlapped with healthy tissue-upregulated genes, supporting the methodological concern that part of the tissue-versus-PBMC signal reflects baseline tissue identity rather than HIV-associated remodeling (**Figure 3B**). To further enrich for residual disease-associated signals, we retained genes whose directional log2 fold-change difference exceeded the healthy tissue-versus-blood background by at least 1. This filtering yielded 1,767 putative HIV-associated upregulated genes and 332 putative HIV-associated downregulated genes (**Figure 3C**). KEGG enrichment analysis of the filtered upregulated genes showed persistent enrichment of PI3K-Akt signaling, cytokine-cytokine receptor interaction, integrin/cell adhesion, ECM-receptor interaction, IL-17 signaling, TNF signaling, and complement/coagulation pathways (**Figure 3D**). These findings indicate that although baseline tissue identity accounts for part of the transcriptomic separation, inflammatory, adhesion/ECM, and PI3K-Akt-related signatures remain highly prominent after healthy-background filtering.

### Rectum and SAT exhibit distinct tissue-specific functional enrichment and expression patterns

**Figure 4:**
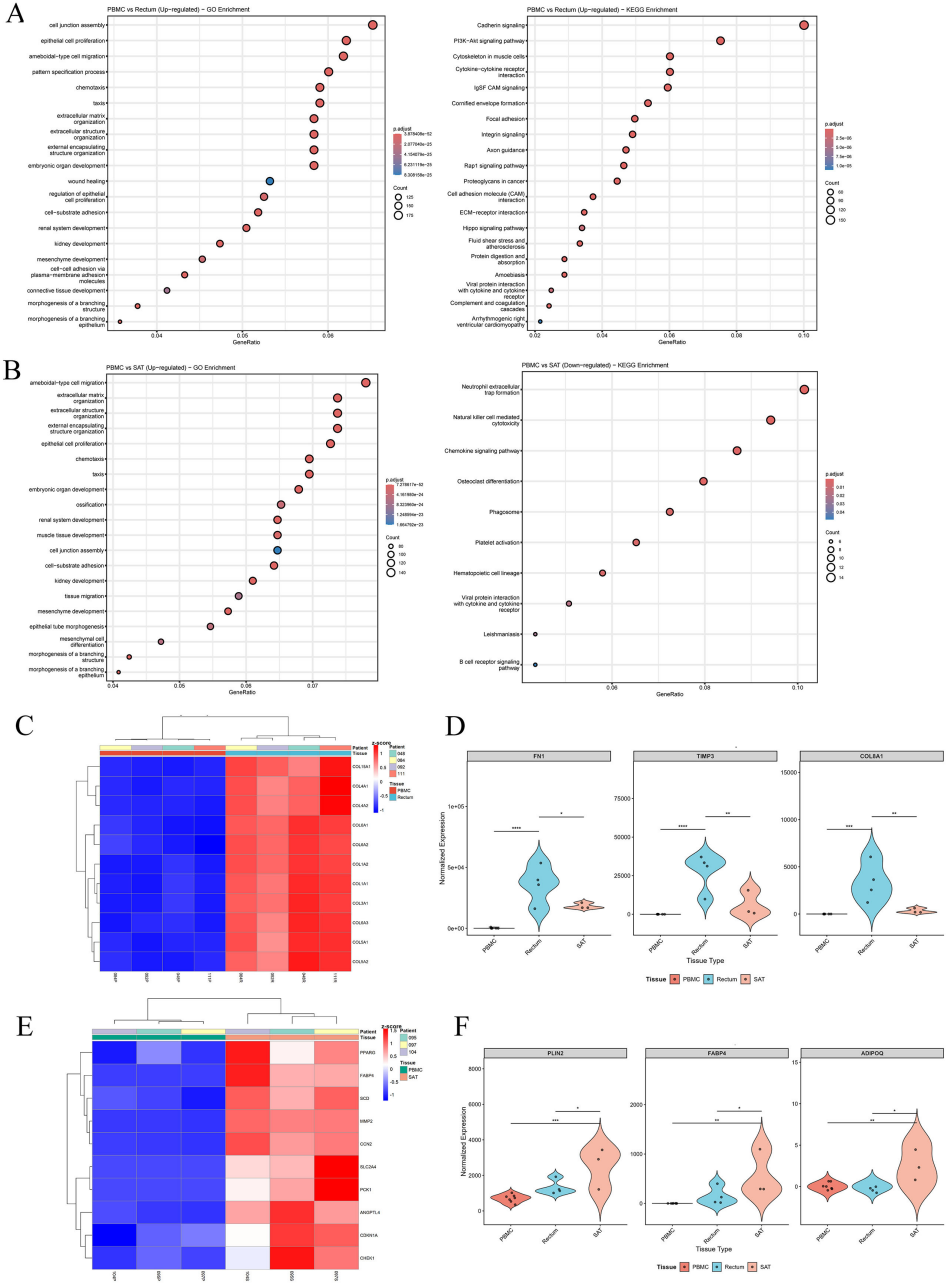
Rectum and SAT exhibit distinct tissue-specific functional enrichment and expression patterns. **(A)** Bubble plots showing GO biological process and KEGG pathway enrichment for genes up-regulated in the rectum. **(B)** Bubble plots showing GO biological process enrichment for up-regulated genes and KEGG enrichment for down-regulated genes in SAT. **(C)** Heatmaps showing the expression of top rectum-specific genes. **(D)** Violin plots showing normalized expression levels of representative tissue-specific markers. **(E)** Heatmaps showing the expression of top SAT-specific genes. **(F)** Violin plots showing normalized expression levels of representative tissue-specific markers. Data are presented as mean ± SEM. Statistical significance was determined by one-way ANOVA with Tukey’s *post hoc* test. *P < 0.05, **P < 0.01, ***P < 0.001, and ****P < 0.0001.

To define the biological programs characterizing these sanctuaries after accounting for tissue background, we further analyzed the specific functional profiles and representative signatures validated in our cohorts. In the Rectum vs. PBMC comparison, GO biological process and KEGG analyses of upregulated genes revealed a marked enrichment in pathways governing ECM remodeling and cell junction assembly (**Figure 4A**). Specifically, GO terms such as extracellular matrix organization and cell junction assembly were highly prominent, while KEGG analysis further identified ECM-receptor interaction and Adhesion-related pathways. These signatures indicate active stromal remodeling and enhanced structural organization within the rectal reservoir relative to PBMCs.

In the SAT vs. PBMC comparison, upregulated genes were significantly enriched in cell cycle-related biological processes, whereas downregulated genes were associated with the Phagosome and immune-related signaling (**Figure 4B**). These data suggest that, compared with peripheral blood, the adipose reservoir is characterized by heightened metabolic and proliferative activity, coupled with a relative suppression of phagocytosis-mediated innate immune surveillance.

We further visualized representative tissue-specific signatures using heatmaps and violin plots to confirm these expression programs. In rectal tissue, multiple collagen family members and ECM-associated genes showed consistently high expression, effectively segregating rectal samples from both PBMC and SAT groups (**Figure 4C**). Violin plots of representative genes, including *FN1, TIMP3*, and *COL1A1*, confirmed their highest expression levels in rectum (**Figure 4D**). Conversely, genes involved in metabolic regulation and adipocyte-related functions (e.g., *ADIPOQ*, *FABP4*) were preferentially expressed in SAT, showing minimal expression in matched PBMCs and rectal tissues (**Figures 4E, F**). Collectively, these findings underscore divergent transcriptional programs between anatomical sanctuaries, with the rectum defined by structural/fibrotic remodeling and the SAT characterized by an immunometabolic and proliferative molecular landscape.

### Common HIV-specific DEGs reveal shared PI3K-Akt and adhesion-related molecular features in the rectum and SAT

**Figure 5:**
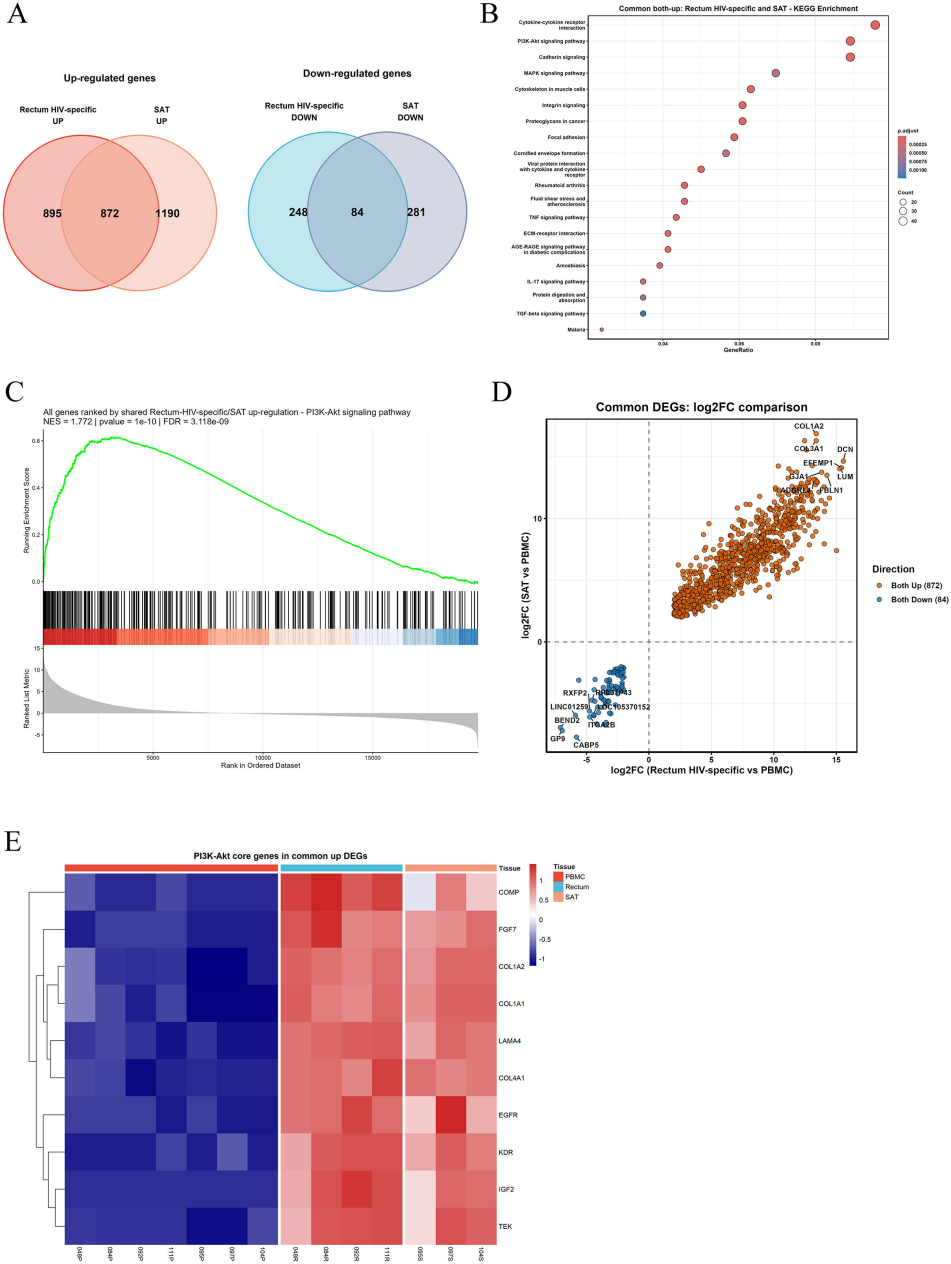
Common DEGs reveal shared PI3K-Akt and adhesion-related molecular features in the rectum and SAT. **(A)** Venn diagrams showing overlapping up-regulated and down-regulated genes between the rectum HIV-specific and SAT DEG sets after healthy-background filtering. **(B)** KEGG enrichment analysis for commonly up-regulated genes. Dot size represents gene count and color indicates adjusted P value. **(C)** GSEA plot demonstrating significant enrichment of the PI3K-Akt signaling pathway in the shared up-regulated gene set (NES = 1.772, pvalue = 1e-10, FDR = 3.118e-09). **(D)** Scatter plot illustrating the correlation of log2 fold changes for common DEGs between the rectum and SAT, indicating highly concordant expression patterns. Each point represents one gene; orange and blue dots indicate both-up-regulated (n = 872 genes) and both-down-regulated (n = 84 genes), respectively. **(E)** Heatmap showing the expression profiles of core PI3K-Akt signaling genes across PBMCs, rectum, and SAT.

To define the molecular programs commonly shared across distinct anatomical sanctuaries after accounting for tissue-specific backgrounds, we implemented a secondary cross-tissue intersection strategy. Given that healthy reference datasets are publicly available for the gut but limited for matched subcutaneous adipose tissue, we intersected the background-filtered rectal reservoir dataset with the core responsive signatures from the SAT compartments (**Figure 5A**). This intersecting matrix identified 872 consistently upregulated genes and 84 consistently downregulated genes that successfully bypassed discordant tissue-specific identities, capturing a refined transcriptomic signature directly associated with persistent viral sequestration.

Functional pathway enrichment analysis of these core intersected genes revealed a prominent overrepresentation of the PI3K-Akt signaling pathway and Focal adhesion, alongside multiple corporate cell-matrix interactive programs, including ECM-receptor interaction and corporate adhesion pathways (**Figure 5B**). This reinforces a coordinated transcriptional program of altered adhesion- and ECM-related remodeling across both tissue reservoirs relative to the peripheral circulation.

To further confirm the global shift of these pathways, all detectable genes were ranked by their shared cross-tissue upregulation and subjected to preranked KEGG Gene Set Enrichment Analysis (GSEA). This analysis revealed a robust, pathway-wide positive enrichment of the PI3K-Akt signaling axis (NES = 1.772, adjusted P < 0.0001) (**Figure 5C**). These results indicate that PI3K-Akt-related gene enrichment is not merely driven by a few highly differential genes but reflects a coordinated transcriptional upward shift across both tissue compartments.

The quantitative concordance of these transcriptional changes was evaluated by comparing the *log_2_* fold changes of the shared genes between the rectal and adipose reservoir datasets. The resulting scatter plot demonstrated that the majority of common DEGs clustered tightly in the first and third quadrants, representing genes with highly consistent directions of change in both tissue compartments (**Figure 5D**), with commonly upregulated genes being markedly predominant. Visualization of core components via expression heatmap further illustrated their consistently elevated expression in both tissue reservoirs compared to matched PBMCs, highlighting key shared structural and signaling elements within this convergent dataset (e.g., *COL1A1*, *COL4A1*, *EGFR*, and *KDR*) (**Figure 5E**). Together, these findings identify the PI3K-Akt-adhesion axis as a potential shared molecular feature accompanying tissue-level host remodeling and persistent HIV-1 sequestration.

### PPI network analysis identifies key hub genes and functional modules within the shared transcriptional program

**Figure 6:**
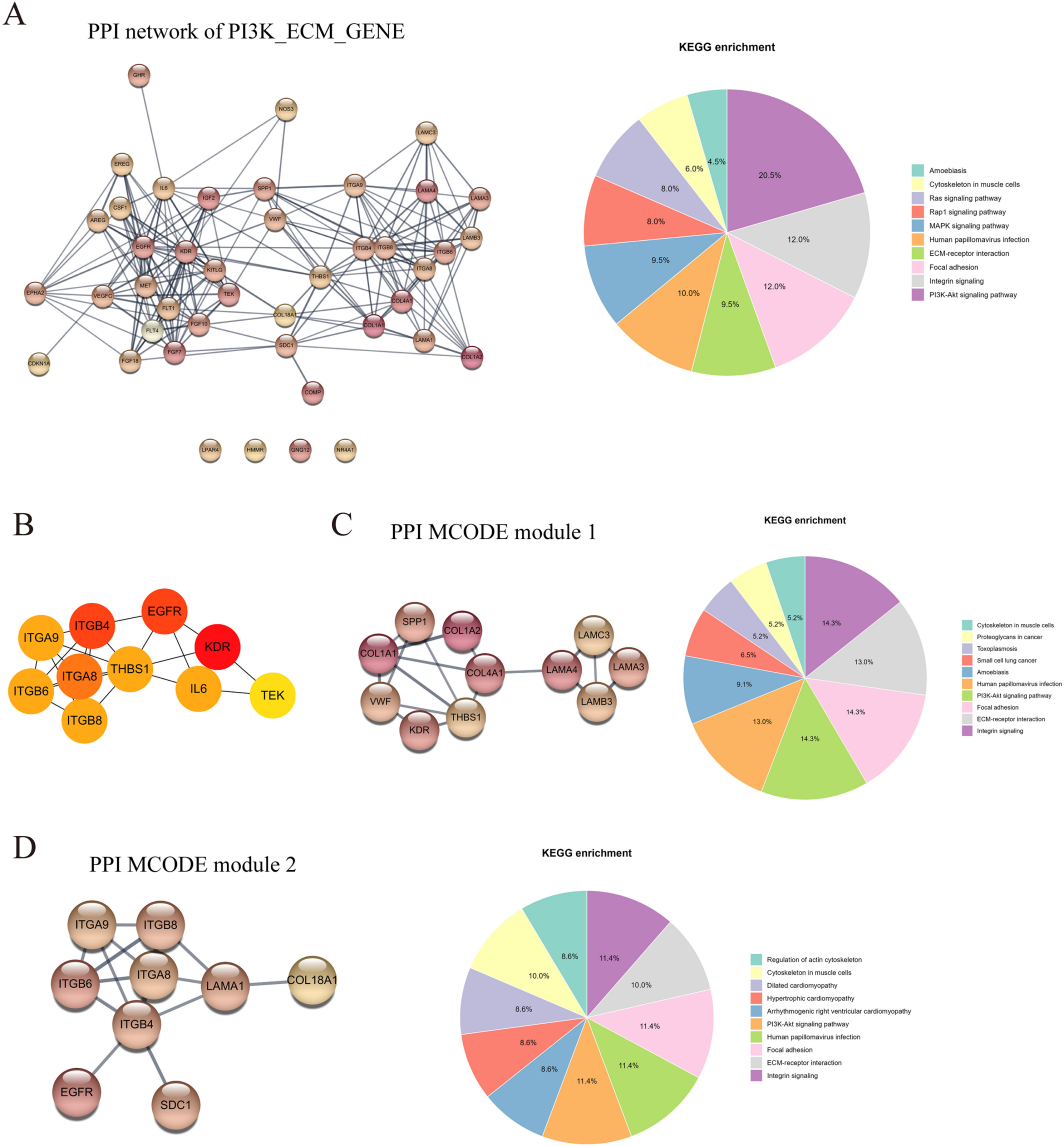
PPI network analysis identifies key hub genes and functional modules among commonly up-regulated genes. **(A)** PPI network constructed from commonly up-regulated genes associated with the PI3K-Akt and ECM-related pathways, with KEGG enrichment shown on the right. Node color intensity represents the mean log_2_ fold change across both tissues. **(B)** Identification of the top 10 hub genes using the CytoHubba degree algorithm. **(C, D)** Functional modules identified by the MCODE algorithm. Module 1 **(C)** and Module 2 **(D)** are shown alongside their respective KEGG enrichment results.

To elucidate the functional interplay among the commonly upregulated genes, we constructed a protein-protein interaction (PPI) network focusing on those associated with the PI3K-Akt and ECM-related pathways. The resulting network exhibited a highly interconnected topology, suggesting that these genes do not function in isolation but instead form a tightly orchestrated signaling and matrix-regulatory interactome (**Figure 6A**). Node color intensity, mapping the mean *log_2_* fold change across rectal and adipose tissues, highlighted several ECM components, integrin family members, and growth factor receptors as high-intensity nodes, underscoring their pivotal roles in shared tissue remodeling.

We next identified the central regulatory nodes within this network using the CytoHubba degree algorithm (32). The top 10 hub genes, including *ITGA9, ITGB4, EGFR, KDR, ITGB6, ITGA8, THBS1, IL6, ITGB8, and TEK*, occupied central positions with exceptionally high connectivity (**Figure 6B**). The hub ranking, visualized by a color gradient from yellow to red, suggests that these integrin- and receptor-associated nodes serve as core mediators integrating PI3K-Akt signaling with cell-adhesion and ECM organization.

Furthermore, MCODE clustering analysis identified two major functional modules within the PPI landscape (33). Module 1 was predominantly composed of structural ECM and cell-adhesion genes, representing a conserved ECM-adhesion subnetwork (**Figure 6C**). KEGG enrichment of this module further highlighted ECM-receptor interaction, Focal adhesion, PI3K-Akt signaling pathway, and Cytoskeleton in muscle cells. Module 2 centered on integrin/receptor-associated genes, indicating a distinct adhesion- and receptor-driven signaling hub (**Figure 6D**). KEGG enrichment of this module showed prominent involvement of the PI3K-Akt signaling pathway, regulation of actin cytoskeleton, cardiomyopathy-related pathways, Focal adhesion, ECM-receptor interaction, and Integrin signaling. Collectively, these structural insights demonstrate that the shared host transcriptomic profiles associated with HIV-1 persistence are organized into discrete yet interconnected functional subnetworks, dedicated to ECM structural integrity and transmembrane receptor signaling.

### Validation of site-specific hub signatures and shared signaling dysregulation

**Figure 7:**
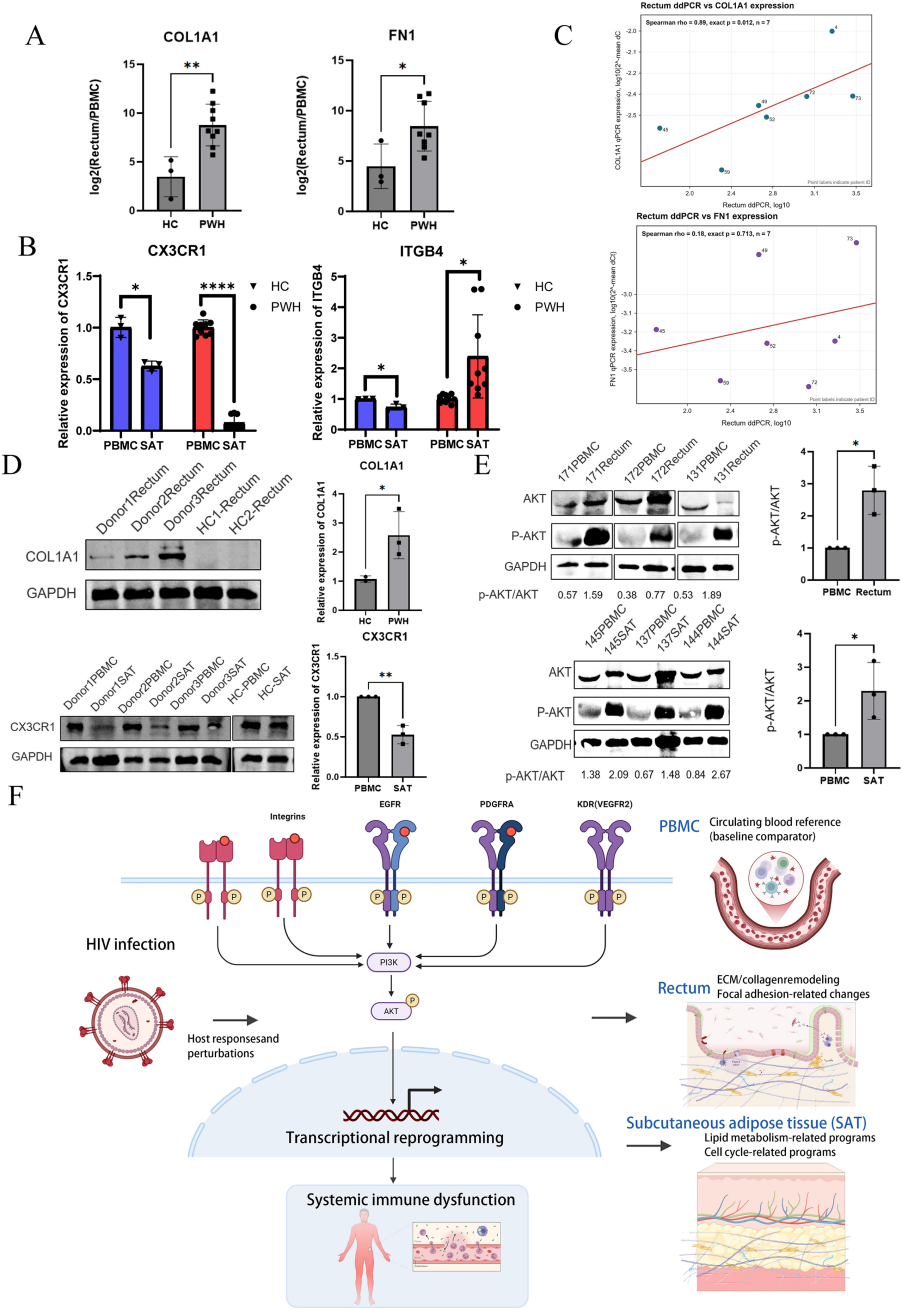
Validation of key molecular changes, reservoir-expression correlations, and PI3K/Akt-related tissue remodeling. **(A)** RT-qPCR validation of fibrosis-related genes (*COL1A1* and *FN1*) in rectal tissue compared with PBMCs. Data are presented as log_2_-transformed ratios. **(B)** RT-qPCR validation of *CX3CR1* and *ITGB4* expression normalized to TBP. CX3CR1 is shown as log2 (PBMC/SAT) in HC and PWH, and ITGB4 is shown as log2(SAT/PBMC) in HC and PWH. **(C)** Spearman correlation between rectal ddPCR values (log10) and rectal qPCR expression of *COL1A1* or *FN1* [log10(2^-mean dCt)] in PWH (n = 7). Point labels indicate patient IDs. **(D)** Western blot analysis of CX3CR1 and COL1A1 protein levels. Representative blots (left) and densitometric quantification (right) are shown. **(E)** Evaluation of PI3K/Akt pathway activation by Western blot. The ratio of p-Akt (Ser473) to total Akt was used to represent pathway activation. GAPDH served as the loading control. **(F)** Schematic model illustrating HIV-associated PI3K/Akt-related transcriptional reprogramming and tissue-specific remodeling. Individual points represent biological samples. Statistical significance in **(A, B, D, E)** was determined using unpaired Student’s t-test; correlations in **(C)** were assessed using Spearman rank correlation. *P < 0.05, and **P < 0.01.

To validate the transcriptomic architectures parsed through our bioinformatic workflow, we performed targeted RT-qPCR validation on paired tissue and PBMC samples from people with HIV (PWH) and healthy controls (HC). We first assessed the major site-specific regulatory expressions defining the rectal microenvironment. Here, we selected the top-ranked shared hub *COL1A1* alongside *FN1*, which was identified in our earlier filtering workflow as a highly prominent, rectum-specific fibrotic driver (**Figure 7A**). Consistent with the tissue profiling, these fibrosis-associated candidates exhibited marked, significant upregulation in the rectal tissues of PWH relative to matched PBMCs. These data, presented as *log_2_*-transformed ratios, confirm a localized pro-fibrotic remodeling program characterizing the mucosal sanctuary during chronic suppressive ART.

In parallel, we examined the distinctive molecular signatures characterizing the adipose sanctuary (**Figure 7B**). In the SAT compartments, where transcripts were strictly normalized to the adipose-stable housekeeping gene *TBP*, we evaluated the tissue-specific adhesion hub *ITGB4* alongside the pivotal immunometabolic/trafficking receptor *CX3CR1*. *CX3CR1* exhibited a significantly increased log2(PBMC/SAT) ratio in PWH compared with HC, suggesting a localized suppression or altered dynamics of this myeloid trafficking axis within the lipid-rich reservoir. Conversely, the cell-matrix adhesion node *ITGB4* demonstrated a prominent, dedicated increase from PBMC to SAT specifically in PWH donors. Together, these paired patterns confirm that while the rectum is governed by structural collagen deposition, the adipose sanctuary is independently verified by distinct adhesion- and immunometabolic-related transcriptomic programs.

To bridge these individual site-specific profiles with local viral persistence and shared downstream cascades, we next evaluated their correlation with the tissue proviral burden and protein-level pathway kinetics. Spearman rank analysis revealed that rectal *COL1A1* expression shared a strong, statistically significant positive correlation with local intact HIV-1 DNA levels quantified by ddPCR (**Figure 7C**), directly connecting host structural remodeling with mucosal reservoir size.

Finally, to validate these altered programs at the functional translation level, Western blot analysis was conducted to measure core cascade components and the activation status of the shared PI3K-Akt pathway (**Figures 7D, E**). In complete alignment with the mRNA patterns, COL1A1 protein levels were markedly elevated in rectal tissues from PWH, whereas CX3CR1 protein expression exhibited a corresponding tissue-specific deficit in SAT compartments. Crucially, evaluating the downstream signaling core via the phosphorylation of Akt at Ser473 (p-Akt/Akt ratio) demonstrated a profound, cross-tissue dysregulation of the PI3K-Akt signaling axis in PWH donors relative to HCs.

In summary, our validation data support a model in which persistent HIV-1 sequestration is accompanied by tissue-specific immune remodeling. Specifically, we propose that HIV-associated transcriptomic reprogramming, which shares features with the PI3K/Akt axis, coincides with structural fibrosis in the rectum and altered immune trafficking and metabolic signaling in adipose tissue, collectively characterizing the maintenance of the systemic viral reservoir and host immune dysfunction (**Figure 7F**).

## Discussion

The persistent existence of HIV-1 reservoirs in anatomical sanctuaries remains the primary obstacle to a functional cure (1, 34). In the present study, we utilized an integrated approach combining high-sensitivity ddPCR and transcriptomic profiling to delineate the host molecular landscape of rectal and adipose reservoirs relative to matched PBMCs. By implementing an external healthy-background filtering strategy, our data demonstrate that while both the rectum and SAT harbor significantly higher viral burdens than peripheral blood, they exhibit divergent transcriptomic programs, characterized by structural remodeling in the rectum and immunometabolic shifts in the SAT. Crucially, we identified the PI3K-Akt signaling axis as a shared molecular feature across these distinct sites, suggesting a common host-response signature parallel to persistent viral sequestration.

The gastrointestinal tract, specifically the rectum, has long been recognized as a major site of HIV-1 persistence and early mucosal CD4^+^ T cell depletion (17, 35). Our findings that rectal tissue undergoes extensive ECM reorganization and cell junction assembly align with the fibrotic sanctuary model of viral persistence. The significant upregulation of collagen family members (e.g., *COL1A1*) and *FN1*, validated at both mRNA and protein levels, suggests that chronic viral sequestration coincides with a pro-fibrotic environment. This structural remodeling not only impairs mucosal barrier integrity—potentially exacerbating microbial translocation—but may also physically shield latent reservoirs from immune surveillance and drug penetration, further complicating eradication efforts (36, 37).

A key highlight of this study is the characterization of the adipose reservoir as an immunometabolic sanctuary (23). Unlike the structural focus of the rectal reservoir, the SAT transcriptomic profile was dominated by cell cycle dysregulation and metabolic activation (38). The observed reduction in *CX3CR1* expression in SAT suggests an impairment in leukocyte recruitment or altered myeloid cell dynamics within the lipid-rich microenvironment. Given that adipose tissue is an active endocrine organ, its role in sequestering HIV-1 may involve a unique interplay between viral persistence and host metabolic signaling (27). SAT is also highly vascularized and represents a large anatomical compartment, which may provide an expanded tissue space for viral persistence. In addition, the lipid-rich adipose microenvironment may influence antiretroviral drug distribution and local exposure via lipophilic partitioning dynamics, potentially limiting the spatial bio-availability of ART around resident target cells and thereby contributing to the higher proviral DNA burden observed in SAT (39). The suppression of phagosome-related pathways further indicates a localized immune-evasive environment that favors viral longevity, which is phenotypically distinct from the patterns observed in mucosal or lymphoid tissues.

One notable finding of our study is the identification of the PI3K-Akt signaling pathway as a shared pathway associated with host responses in both the rectum and SAT. PI3K-Akt is a master regulator of cell survival, metabolism, and structural remodeling (40). In the context of HIV-1, this pathway has been implicated in maintaining viral latency and promoting the survival of infected cells. Our GSEA and PPI network analyses indicate that PI3K-Akt-related enrichment may represent a shared feature of tissue reservoirs. We hypothesize that PI3K-Akt signaling may act as a molecular bridge: in the rectum, it may be linked to fibrotic responses through integrin-mediated signaling, while in the SAT, it may be associated with metabolic survival programs in the reservoir. However, these transcriptomic and network-based findings support association rather than causality, and functional validation will be required. These observations suggest that the PI3K-Akt axis may be worth exploring as a potential target for “block and lock” strategies aimed at silencing the host environment that supports persistence.

Despite the strengths of our matched multi-tissue analysis, several limitations should be acknowledged. First, our discovery transcriptomic cohort is inherently constrained by a limited sample size (n = 4 paired rectal samples and n = 3 paired SAT samples), which may restrict the generalization of the identified transcriptomic repertoire. Although we integrated a strictly paired multi-factor statistical framework within DESeq2 to control for inter-individual biological variance and applied highly stringent filtering thresholds (adjusted P < 0.01, *log_2_* FC > 2), replication in larger, multi-center cohorts is fundamentally required to validate the stability of these signatures. Second, due to the absolute logistical and ethical constraints in obtaining paired healthy tissue biopsies from uninfected individuals concurrently, our primary discovery workflow lacked internal HIV-negative tissue controls. While we successfully implemented an independent cross-dataset background subtraction strategy using healthy reference matrices to strip away baseline tissue-identity noise, future prospective studies encompassing concurrent, site-matched healthy tissue controls remain necessary to fully disentangle universal tissue homeostatic baselines from genuine HIV-induced pathological remodeling. Concurrently, due to the extreme tissue-volume limitations of these microscopic clinical biopsies, parallel DNA and RNA extractions could not be performed for identical SAT samples, which mathematically precluded intra-individual correlation analyses within the adipose compartment. Third, our reliance on bulk RNA-sequencing provides a global microenvironmental landscape but inevitably obscures cell-type-specific contributions. Consequently, we could not computationally partition whether the altered transcriptomic features stem directly from latent viral hosts (such as CD4^+^ T cells and macrophages) or from neighboring parenchymal components (such as adipocytes) that shape the metabolic sanctuary (41, 42). Future studies utilizing scRNA-seq or spatial transcriptomics are needed to dissect these cell-specific interactions (43, 44). Finally, the mechanistic interpretation of the PI3K-Akt pathway in this study remains characteristically correlative; formal functional inhibition studies in automated organoid models or humanized mice will be necessary to confirm the precise, causal role of this signaling axis in host microenvironment reprogramming and viral persistence.

In conclusion, our study provides a high-resolution map of the transcriptomic remodeling that occurs within anatomical HIV-1 reservoirs during suppressive ART. By identifying both the divergent site-specific responses and the shared PI3K-Akt signaling core, we underscore the necessity of moving beyond a “one-size-fits-all” approach to HIV-1 cure research. Targeting the tissue-specific microenvironments—particularly the structural barriers in the gut and the immunometabolic shields in adipose tissue—will be essential for successfully disrupting the persistence of the viral reservoir.

## Materials and methods

### Participants

Peripheral blood for the isolation of primary CD4^+^ T cells was obtained from HIV-1-infected individuals. HIV-1-infected individuals had been on ART for at least 12 months and had maintained undetectable HIV-1 viremia (<50 HIV-1 RNA copies per ml of plasma). All the HIV-1-infected individuals were recruited from Shenzhen Third People’s Hospital (**Supplementary Table 1**). Buffy coats derived from the blood of healthy donors were used in *in vitro* experiments.

### Sample processing

Rectum and SAT specimens were placed on ice immediately after collection and thoroughly rinsed with pre-chilled PBS to remove surface debris and residual blood. The tissues were then trimmed to remove cauterized areas and residual blood clots, and cut into appropriately sized fragments according to downstream experimental requirements. For transcriptome sequencing, tissue samples were finely minced in tissue dissociation buffer. The digestion buffer was prepared in DPBS containing *Ca²+/Mg²+* and typically consisted of Collagenase IV (0.5–2.0 mg/mL), Dispase II (1.0–2.0 mg/mL), Hyaluronidase (0.1–1.0 mg/mL), and DNase I (20–50 μg/mL). The minced tissues were transferred to 15 mL centrifuge tubes, mixed with an appropriate volume of digestion buffer, and enzymatically digested in a 37 °C water bath for 35–50 min. The digestion products were filtered through a 70 μm cell strainer, washed with PBS, and centrifuged at 500 g for 5 min at 4 °C. After removal of the supernatant, red blood cell lysis buffer was added for 5 min, followed by centrifugation; if obvious RBC contamination remained, the lysis step was repeated. The final cell pellets were directly lysed in TRIzol reagent, stored at -80 °C, and submitted to BGI (Shenzhen, China) for DNBSEQ eukaryotic transcriptome sequencing. Tissue samples used for subsequent protein and nucleic acid analyses were snap-frozen in liquid nitrogen and stored at -80 °C. For Western blot and qPCR experiments, frozen tissues were placed on ice and homogenized using a handheld homogenizer in either TRIzol reagent or NP40 protein lysis buffer supplemented with protease inhibitors for total RNA or protein extraction.

Peripheral blood samples were first centrifuged at 2000 rpm for 5 min, and the upper plasma layer was collected and stored. PBS was then added to the remaining lower phase and mixed gently, after which the suspension was slowly layered onto an equal volume of Ficoll-Paque. Density gradient centrifugation was performed at 1000 × g for 15 min using the lowest acceleration and deceleration settings, and the mononuclear cell layer was collected. The cells were washed with PBS, treated with red blood cell lysis buffer to remove residual erythrocytes, and centrifuged to obtain cell pellets. The cells were then resuspended in freezing medium prepared with FBS/DMSO (9:1) and cryopreserved by gradient freezing at -80 °C. For transcriptome sequencing, PBMC pellets were lysed in TRIzol reagent and stored before sequencing.

### RNA-seq data preprocessing

Raw sequencing data from 14 paired-end bulk RNA-seq samples were first assessed using FastQC (v0.12.1). Raw FASTQ files were then processed with fastp (v0.23.2) for adapter trimming and quality control using the parameters --detect_adapter_for_pe, --trim_poly_g, and --cut_right, and the filtered reads were written to the cleandata directory. Clean reads were then aligned to the human reference genome GRCh38 primary assembly using HISAT2 (v2.2.1). The alignment index was built from Homo_sapiens.GRCh38.dna.primary_assembly.fa, and gene annotation was based on Ensembl release 109 (Homo_sapiens.GRCh38.109.gtf.gz). The resulting SAM files were converted to BAM format and sorted using samtools (v1.9). Gene-level read counts were generated using featureCounts (v2.0.1) with paired-end counting at the exon level and summarized by gene_id. Count files from all samples were subsequently merged into a gene expression matrix for downstream analysis.

### Differential expression and transcriptomic analyses

All downstream analyses were performed locally in R (v4.3.3). Raw count data were imported into R, and Ensembl gene IDs were converted to gene symbols using org.Hs.eg.db after removal of Ensembl version suffixes. Genes without valid gene symbols were excluded. For duplicated entries mapping to the same gene symbol, expression values were averaged and rounded to generate a gene-level count matrix.

Principal component analysis (PCA) was performed on variance-stabilized expression data generated by DESeq2 after size-factor normalization. Genes with low abundance were filtered using a threshold of total raw counts <10 across the included samples. Variance stabilizing transformation (VST) was performed with blind = TRUE for PCA visualization.

Differential expression analysis was conducted separately for Rectum vs PBMC and SAT vs PBMC using DESeq2 with paired designs (~ patient + tissue) to account for inter-individual variation. The Rectum vs PBMC comparison included four paired samples, whereas the SAT vs PBMC comparison included three paired samples. Genes with rowSums(counts) < 10 were removed before model fitting. Differentially expressed genes (DEGs) were defined as those with an adjusted P value <0.01 and an absolute *log_2_* fold change >2. Positive *log_2_* fold changes indicated higher expression in rectum or SAT relative to PBMC, whereas negative values indicated higher expression in PBMC.

### Functional enrichment, overlap analysis, and visualization

GO biological process enrichment analysis was performed using clusterProfiler::enrichGO with keyType = “SYMBOL”, ont = “BP”, Benjamini-Hochberg correction, pvalueCutoff = 0.05, and qvalueCutoff = 0.2. KEGG pathway enrichment analysis was carried out using clusterProfiler::enrichKEGG after conversion of gene symbols to Entrez IDs, with organism = “hsa” and pvalueCutoff = 0.05.

To compare transcriptional overlap between Rectum vs. PBMC and SAT vs. PBMC, DEGs from the two comparisons were intersected and classified into rectum-specific, SAT-specific, and common gene sets. Common genes were further subdivided into both-upregulated, both-downregulated, and oppositely changed categories according to the direction of differential expression.

Healthy-background filtering was performed using the external normal human tissue dataset GSE120795. Healthy colon/colon epithelium samples were used as the tissue reference, and whole-blood nuclear-cell samples were used as the blood reference. HIV tissue-versus-PBMC DEGs were retained as putative HIV-associated genes when their directional tissue-versus-PBMC log2 fold-change exceeded the corresponding healthy tissue-versus-blood background by at least 1.

For GSEA of the common gene set, a custom ranking score was calculated for each shared gene as:

Score = sign (log_2_FC_Rectum_ + log_2_FC_SAT_) × min (|log_2_FC_Rectum_|, |log_2_FC_SAT_|)

thereby prioritizing genes with concordant changes in both tissue comparisons. Ranked genes were analyzed using clusterProfiler::gseKEGG with organism = “hsa”, minGSSize = 10, maxGSSize = 500, and pvalueCutoff = 1.The PI3K-Akt signaling pathway was specifically extracted for visualization, and the normalized enrichment score (NES) together with nominal P value and FDR were displayed on the GSEA plot.

Heatmaps were generated using pheatmap. For pairwise heatmaps, VST-transformed expression values from the corresponding DESeq2 objects were used, followed by row-wise z-score scaling. For the common PI3K-Akt core-gene heatmap across PBMC, rectum, and SAT, normalized counts were transformed as log2(normalized count + 1) and then standardized by row-wise z-score. Violin plots were generated from size-factor-normalized counts. Candidate tissue-specific genes were screened by comparing mean normalized expression across PBMC, rectum, and SAT, and only genes showing the highest mean expression in the target tissue were retained for plotting.

### Protein-protein interaction network analysis

Genes shared by the PI3K-Akt signaling pathway and ECM-receptor interaction pathway were extracted from the common upregulated gene set, deduplicated, and assigned an average log_2_ fold change calculated as the mean of the Rectum vs PBMC and SAT vs PBMC log_2_ fold changes. These genes were imported into Cytoscape (v3.10.4) for protein-protein interaction (PPI) network analysis. The STRING network was constructed using the full STRING network option, with a confidence score cutoff of 0.70 and maximum additional interactors set to 0. Smart delimiters were not used, whereas enrichment data loading was enabled. Node color intensity was mapped to the average log_2_ fold change. Hub genes were ranked using the Degree algorithm implemented in cytoHubba (v0.1), and the top 10 genes were selected as hub genes (32). Network modules were identified using the MCODE algorithm in clusterMaker2 (v2.3.4) with the following parameters: degree cutoff = 2, node score cutoff = 0.2, k-core = 2, and max depth = 100; haircut was enabled, whereas fluff and loop inclusion were disabled (33). A new clustered network was generated for visualization, and inter-cluster edges were not restored after layout. The two major modules were selected for presentation.

### Western blotting

PBMCs, rectal tissue, and SAT were collected and lysed using NP-40 lysis buffer (10 mM Tris-HCl, pH 7.5, 150 mM NaCl, 1% NP-40, 1% Triton X-100, 10% glycerol, 0.2 mM EDTA) supplemented with protease inhibitor cocktail and phosphatase inhibitors (TOPSCIENCE, Shanghai, China). Tissue samples were mechanically homogenized prior to lysis. All samples were incubated on ice for 30 min with vortexing every 10 min.

The lysates were clarified by centrifugation at 12,000 × g for 5 min at 4 °C, and the supernatants were collected as total protein extracts. Protein concentrations were determined using a BCA protein assay kit (Vazyme Biotech Co., Ltd., Nanjing, China) according to the manufacturer’s instructions. Equal amounts of protein were mixed with loading buffer containing dithiothreitol (DTT), boiled at 100 °C for 10 min, separated by SDS-PAGE, and transferred onto nitrocellulose membranes (PALL, Port Washington, NY, USA). Membranes were blocked with Protein-Free Rapid Blocking Buffer (1x; Epizyme, PS108P) for 1 h at room temperature and incubated overnight at 4 °C with primary antibodies: anti-p-Akt (Ser473; StarterBio, S0B0783, mouse mAb clone S-769-59A), anti-Akt (pan; StarterBio, S0B0114, recombinant rabbit mAb clone SDT-R169), anti-Collagen I (COL1A1; StarterBio, S0B6036, recombinant rabbit mAb clone S-1473-5), anti-CX3CR1 (Proteintech, 29819-1-AP, rabbit polyclonal), and anti-GAPDH.

After washing with TBST, membranes were incubated with IRDye-conjugated secondary antibodies (LI-COR, Lincoln, NE, USA) for 1 h at room temperature. Protein bands were visualized using the Odyssey infrared imaging system (LI-COR). Band intensities were quantified using ImageJ software (NIH, Bethesda, MD, USA). The expression levels of target proteins were normalized to GAPDH. The activation of the PI3K/Akt pathway was determined by the ratio of p-Akt (Ser473) to total Akt.

### RT-qPCR analysis

Total RNA was extracted from PBMCs, rectal tissue, and SAT using TRIzol reagent according to the manufacturer’s instructions. RNA concentration and purity were assessed prior to reverse transcription. Complementary DNA (cDNA) was synthesized using a reverse transcription kit (Vazyme Biotech Co., Ltd., Nanjing, China) following the manufacturer’s protocol. Quantitative real-time PCR (qPCR) was performed using a SYBR Green qPCR kit (Vazyme) on a QuantStudio 3 Real-Time PCR System (Applied Biosystems, USA). Relative gene expression levels were calculated using the 2^-ΔΔCt^ method and normalized to the internal control gene GAPDH or TBP, as indicated in the corresponding figure panel. Each sample was analyzed using at least three independent biological replicates (n ≥ 3 donors).

For fibrosis-related gene validation, *COL1A1* and *FN1* expression levels were measured in rectal tissue and PBMC groups. For chemotaxis- and adhesion-related gene validation, *CX3CR1* and *ITGB4* expression levels were assessed in subcutaneous fat and PBMC groups. In **Figure 7B**, CX3CR1 was presented as log2(PBMC/SAT), whereas ITGB4 was presented as log2(SAT/PBMC).

The primer sequences used in this study were as follows:

*COL1A1:* Forward: GTGCGATGACGTGATCTGTGA, Reverse: CGGTGGTTTCTTGGTCGGT
*FN1:* Forward: CGGTGGCTGTCAGTCAAAG, Reverse: AAACCTCGGCTTCCTCCATAA
*CX3CR1:* Forward: AGTGTCACCGACATTTACCTCC, Reverse: AAGGCGGTAGTGAATTTGCAC
*ITGB4:* Forward: CTCCACCGAGTCAGCCTTC, Reverse: CGGGTAGTCCTGTGTCCTGTA
*GAPDH:* Forward: CTGGGCTACACTGAGCACC, Reverse: AAGTGGTCGTTGAGGGCAATG
*TBP:* Forward: CCACTCACAGACTCTCACAAC, Reverse: CTGCGGTACAATCCCAGAACT

### Measurement of the HIV latent reservoir by intact proviral DNA assay

The HIV latent reservoir was quantified using an IPDA based on ddPCR. Genomic DNA was extracted from patient-derived samples according to the manufacturer’s instructions, with precautions taken to minimize DNA shearing. DNA concentration was measured prior to ddPCR analysis.

The assay employed a dual-probe design targeting two conserved regions of the HIV-1 genome, namely the packaging signal (Ψ) and env, to detect and quantify HIV proviral DNA. ddPCR reactions were prepared in a final volume of 20 μL using ddPCR Supermix for Probes (no dUTP). For each patient sample, 800 ng genomic DNA was added per reaction. J-Lat 8.4 genomic DNA was used as a positive control at 30 ng per reaction, and a no-template control was included in each experiment.

Droplets were generated using the QX200 Droplet Generator (Bio-Rad, USA) and transferred to a 96-well PCR plate, followed by heat sealing. PCR amplification was performed with a heated lid set to 105 °C under the following cycling conditions: 95 °C for 10 min, followed by 45 cycles of 94 °C for 30 s and 52 °C for 1 min, and a final step at 98 °C for 10 min, then held at 4 °C. The ramp rate was set to 2 °C/s. Following amplification, droplets were read using the QX200 Droplet Reader (Bio-Rad), and data were analyzed with QuantaSoft software. Droplets were classified based on fluorescence signals; double-positive droplets (Ψ^+^env^+^) were considered to represent intact proviruses, whereas single-positive droplets were classified as defective proviral species. Absolute HIV-1 DNA copy numbers were calculated using Poisson statistics. The frequency of the HIV latent reservoir was normalized to total input cell equivalents and expressed as copies of intact proviral DNA per million total cells.

### Statistical analysis

All experiments were performed using samples derived from at least three independent donors (n ≥ 3). Data are presented as mean ± SEM. Statistical comparisons among groups were performed using one-way ANOVA followed by Tukey’s *post hoc* test. A *P* value < 0.05 was considered statistically significant. Software and packages used in this study were listed in **Supplementary Table 2**. Paired t-tests were used for **Figure 1** ddPCR comparisons, whereas RT-qPCR and Western blot validation assays were analyzed using unpaired Student’s t-tests. Correlations between rectal ddPCR values and qPCR expression were assessed using Spearman rank correlation with exact P values.

## Data Availability

The datasets presented in this study have been deposited in the NCBI Gene Expression Omnibus (GEO) under accession number GSE336629.

## References

[1] Deeks SG Lewin SR Havlir DV . The end of AIDS: HIV infection as a chronic disease. Lancet. (2013) 382:1525–33. doi: 10.1016/s0140-6736(13)61809-7 24152939 PMC4058441

[2] Gandhi RT Bedimo R Hoy JF Landovitz RJ Smith DM Eaton EF . Antiretroviral drugs for treatment and prevention of HIV infection in adults: 2022 recommendations of the International Antiviral Society-USA Panel. Jama. (2023) 329:63–84. doi: 10.1001/jama.2020.17025 36454551

[3] Siliciano JD Siliciano RF . Recent developments in the effort to cure HIV infection: going beyond N = 1. J Clin Invest. (2016) 126:409–14. doi: 10.1172/jci86047 26829622 PMC4731192

[4] Bachmann N von Siebenthal C Vongrad V Turk T Neumann K Beerenwinkel N . Determinants of HIV-1 reservoir size and long-term dynamics during suppressive ART. Nat Commun. (2019) 10:3193. doi: 10.1038/s41467-019-10884-9 31324762 PMC6642170

[5] Li JZ Etemad B Ahmed H Aga E Bosch RJ Mellors JW . The size of the expressed HIV reservoir predicts timing of viral rebound after treatment interruption. Aids. (2016) 30:343–53. doi: 10.1097/qad.0000000000000953 26588174 PMC4840470

[6] Einkauf KB Osborn MR Gao C Sun W Sun X Lian X . Parallel analysis of transcription, integration, and sequence of single HIV-1 proviruses. Cell. (2022) 185:266–282.e15. doi: 10.1016/j.cell.2021.12.011 35026153 PMC8809251

[7] Collora JA Liu R Pinto-Santini D Ravindra N Ganoza C Lama JR . Single-cell multiomics reveals persistence of HIV-1 in expanded cytotoxic T cell clones. Immunity. (2022) 55:1013–1031.e7. doi: 10.1016/j.immuni.2022.03.004 35320704 PMC9203927

[8] Yeh YJ Ho YC . Shock-and-kill versus block-and-lock: Targeting the fluctuating and heterogeneous HIV-1 gene expression. Proc Natl Acad Sci USA. (2021) 118(16):e2103692118. doi: 10.1073/pnas.2103692118 33758027 PMC8072369

[9] Armani-Tourret M Bone B Tan TS Sun W Bellefroid M Struyve T . Immune targeting of HIV-1 reservoir cells: a path to elimination strategies and cure. Nat Rev Microbiol. (2024) 22:328–44. doi: 10.1038/s41579-024-01010-8 38337034 PMC11131351

[10] Veazey RS DeMaria M Chalifoux LV Shvetz DE Pauley DR Knight HL . Gastrointestinal tract as a major site of CD4+ T cell depletion and viral replication in SIV infection. Science. (1998) 280:427–31. doi: 10.1126/science.280.5362.427 9545219

[11] Chun TW Nickle DC Justement JS Meyers JH Roby G Hallahan CW . Persistence of HIV in gut-associated lymphoid tissue despite long-term antiretroviral therapy. J Infect Dis. (2008) 197:714–20. doi: 10.1086/527324 18260759

[12] De Scheerder MA Vrancken B Dellicour S Schlub T Lee E Shao W . HIV rebound is predominantly fueled by genetically identical viral expansions from diverse reservoirs. Cell Host Microbe. (2019) 26:347–358.e7. doi: 10.1016/j.chom.2019.08.003 31471273 PMC11021134

[13] Lackner AA Mohan M Veazey RS . The gastrointestinal tract and AIDS pathogenesis. Gastroenterology. (2009) 136:1965–78. doi: 10.1053/j.gastro.2008.12.071 19462506 PMC3755960

[14] Lau JSY Lewin SR Telwatte S . HIV and the gut: implications for HIV persistence, immune dysfunction and cure strategies. Front Immunol. (2025) 16:1650852. doi: 10.3389/fimmu.2025.1650852 41050701 PMC12488651

[15] Mudd JC Brenchley JM . Gut mucosal barrier dysfunction, microbial dysbiosis, and their role in HIV-1 disease progression. J Infect Dis. (2016) 214 Suppl 2:S58–66. doi: 10.1093/infdis/jiw258 27625432 PMC5021240

[16] Guadalupe M Reay E Sankaran S Prindiville T Flamm J McNeil A . Severe CD4+ T-cell depletion in gut lymphoid tissue during primary human immunodeficiency virus type 1 infection and substantial delay in restoration following highly active antiretroviral therapy. J Virol. (2003) 77:11708–17. doi: 10.1128/jvi.77.21.11708-11717.2003 14557656 PMC229357

[17] Brenchley JM Schacker TW Ruff LE Price DA Taylor JH Beilman GJ . CD4+ T cell depletion during all stages of HIV disease occurs predominantly in the gastrointestinal tract. J Exp Med. (2004) 200:749–59. doi: 10.1084/jem.20040874 15365096 PMC2211962

[18] Mehandru S Poles MA Tenner-Racz K Horowitz A Hurley A Hogan C . Primary HIV-1 infection is associated with preferential depletion of CD4+ T lymphocytes from effector sites in the gastrointestinal tract. J Exp Med. (2004) 200:761–70. doi: 10.1084/jem.20041196 15365095 PMC2211967

[19] Hunt PW . HIV and inflammation: mechanisms and consequences. Curr HIV/AIDS Rep. (2012) 9:139–47. doi: 10.1007/s11904-012-0118-8 22528766

[20] Klatt NR Funderburg NT Brenchley JM . Microbial translocation, immune activation, and HIV disease. Trends Microbiol. (2013) 21:6–13. doi: 10.1016/j.tim.2012.09.001 23062765 PMC3534808

[21] McGettrick PMC Mallon PWG . HIV and cardiovascular disease: defining the unmeasured risk. Lancet HIV. (2018) 5:e267–9. doi: 10.1016/s2352-3018(18)30061-4 29731408

[22] Couturier J Lewis DE . HIV persistence in adipose tissue reservoirs. Curr HIV/AIDS Rep. (2018) 15:60–71. doi: 10.1007/s11904-018-0378-z 29423731 PMC5876154

[23] Damouche A Lazure T Avettand-Fènoël V Huot N Dejucq-Rainsford N Satie AP . Adipose tissue is a neglected viral reservoir and an inflammatory site during chronic HIV and SIV infection. PloS Pathog. (2015) 11:e1005153. doi: 10.1371/journal.ppat.1005153 26402858 PMC4581628

[24] Damouche A Pourcher G Pourcher V Benoist S Busson E Lataillade JJ . High proportion of PD-1-expressing CD4(+) T cells in adipose tissue constitutes an immunomodulatory microenvironment that may support HIV persistence. Eur J Immunol. (2017) 47:2113–23. doi: 10.1002/eji.201747060 28762530

[25] Koethe JR Hulgan T Niswender K . Adipose tissue and immune function: a review of evidence relevant to HIV infection. J Infect Dis. (2013) 208:1194–201. doi: 10.1093/infdis/jit324 23878320 PMC3778969

[26] Koethe JR Lagathu C Lake JE Domingo P Calmy A Falutz J . HIV and antiretroviral therapy-related fat alterations. Nat Rev Dis Primers. (2020) 6:48. doi: 10.1038/s41572-020-0181-1 32555389

[27] Sáez-Cirión A Sereti I . Immunometabolism and HIV-1 pathogenesis: food for thought. Nat Rev Immunol. (2021) 21:5–19. doi: 10.1038/s41577-020-0381-7 32764670

[28] Bourgeois C Gorwood J Olivo A Le Pelletier L Capeau J Lambotte O . Contribution of adipose tissue to the chronic immune activation and inflammation associated with HIV infection and its treatment. Front Immunol. (2021) 12:670566. doi: 10.3389/fimmu.2021.670566 34220817 PMC8250865

[29] Sun W Gao C Hartana CA Osborn MR Einkauf KB Lian X . Phenotypic signatures of immune selection in HIV-1 reservoir cells. Nature. (2023) 614:309–17. doi: 10.1038/s41586-022-05538-8 36599977 PMC9908552

[30] Bruner KM Wang Z Simonetti FR Bender AM Kwon KJ Sengupta S . A quantitative approach for measuring the reservoir of latent HIV-1 proviruses. Nature. (2019) 566:120–5. doi: 10.1038/s41586-019-0898-8 30700913 PMC6447073

[31] Suntsova M Gaifullin N Allina D Reshetun A Li X Mendeleeva L . Atlas of RNA sequencing profiles for normal human tissues. Sci Data. (2019) 6:36. doi: 10.1038/s41597-019-0043-4 31015567 PMC6478850

[32] Chin CH Chen SH Wu HH Ho CW Ko MT Lin CY . cytoHubba: identifying hub objects and sub-networks from complex interactome. BMC Syst Biol. (2014) 8 Suppl 4:S11. doi: 10.1186/1752-0509-8-s4-s11 25521941 PMC4290687

[33] Bader GD Hogue CW . An automated method for finding molecular complexes in large protein interaction networks. BMC Bioinf. (2003) 4:2. doi: 10.1186/1471-2105-4-2 12525261 PMC149346

[34] Mundlia P Sharma A Kaur J Chaubey B Ranawat P Sood V . From antiretrovirals to curative therapies: Current developments in HIV treatment and prevention. Eur J Med Chem. (2025) 300:118190. doi: 10.1016/j.ejmech.2025.118190 40986988

[35] Mowat AM Agace WW . Regional specialization within the intestinal immune system. Nat Rev Immunol. (2014) 14:667–85. doi: 10.1038/nri3738 25234148

[36] Schacker TW Nguyen PL Beilman GJ Wolinsky S Larson M Reilly C . Collagen deposition in HIV-1 infected lymphatic tissues and T cell homeostasis. J Clin Invest. (2002) 110:1133–9. doi: 10.1172/jci200216413 12393849 PMC150803

[37] Somsouk M Estes JD Deleage C Dunham RM Albright R Inadomi JM . Gut epithelial barrier and systemic inflammation during chronic HIV infection. Aids. (2015) 29:43–51. doi: 10.1097/qad.0000000000000511 25387317 PMC4444362

[38] Ramirez Bustamante CE Agarwal N Cox AR Hartig SM Lake JE Balasubramanyam A . Adipose tissue dysfunction and energy balance paradigms in people living with HIV. Endocr Rev. (2024) 45:190–209. doi: 10.1210/endrev/bnad028 37556371 PMC10911955

[39] Bourgeois C Gorwood J Barrail-Tran A Lagathu C Capeau J Desjardins D . Specific biological features of adipose tissue, and their impact on HIV persistence. Front Microbiol. (2019) 10:2837. doi: 10.3389/fmicb.2019.02837 31921023 PMC6927940

[40] Manning BD Toker A . AKT/PKB signaling: navigating the network. Cell. (2017) 169:381–405. doi: 10.1016/j.cell.2017.04.001 28431241 PMC5546324

[41] Ehrenberg PK Geretz A Volcic M Izumi T Yum LK Waickman A . Single-cell analyses identify monocyte gene expression profiles that influence HIV-1 reservoir size in acutely treated cohorts. Nat Commun. (2025) 16:4975. doi: 10.1038/s41467-025-59833-9 40442100 PMC12122806

[42] Liu X Zhang L Li X Chen L Lu L Yang Y . Single-cell multi-omics profiling uncovers the immune heterogeneity in HIV-infected immunological non-responders. EBioMedicine. (2025) 115:105667. doi: 10.1016/j.ebiom.2025.105667 40184908 PMC12002939

[43] Wang S Zhang Q Hui H Agrawal K Karris MAY Rana TM . An atlas of immune cell exhaustion in HIV-infected individuals revealed by single-cell transcriptomics. Emerg Microbes Infect. (2020) 9:2333–47. doi: 10.1080/22221751.2020.1826361 32954948 PMC7646563

[44] Hu K O'Neil TR Baharlou H Austin PJ Karrasch JF Sarkawt L . The spatial biology of HIV infection. PloS Pathog. (2025) 21:e1012888. doi: 10.1371/journal.ppat.1012888 39854613 PMC11760614

