# Supplementary material for: Integrated transcriptomic profiling of rectal and adipose HIV-1 reservoirs relative to matched PBMCs in ART-treated individuals
Source: Front Immunol. 2026 Jul 7;17:1873668. doi: 10.3389/fimmu.2026.1873668 (PMC13385039; doi:10.3389/fimmu.2026.1873668)
Supplement: Supplementary file 1 [file Table1.docx]

**Table S1. Clinical characteristics of included patients**

| **Patient ID** | **Sex** | **cART duration** | **Current CD4+ T-cell count, cells/uL** | **cART regimen** |
| --- | --- | --- | --- | --- |
| **4** | **male** | **5years** | **812** | **Bictegravir 50 mg + emtricitabine 200 mg + tenofovir alafenamide 25 mg** |
| **37** | **male** | **10years** | **323** | **Elvitegravir 150 mg + cobicistat 150 mg + emtricitabine 200 mg + tenofovir alafenamide 10 mg** |
| **42** | **male** | **10years** | **833** | **Zidovudine-lamivudine tablet 450 mg + lopinavir-ritonavir tablet 500 mg** |
| **43** | **male** | **6years** | **377** | **Efavirenz 400 mg + lamivudine 300 mg + tenofovir disoproxil fumarate 300 mg qd** |
| **45** | **male** | **10years** | **806** | **Dolutegravir 50 mg + lamivudine 300 mg** |
| **48** | **male** | **2years** | **422** | **Efavirenz 400 mg + lamivudine 300 mg + tenofovir disoproxil fumarate 300 mg qd** |
| **49** | **male** | **7years** | **542** | **Rilpivirine 25 mg + lamivudine 300 mg + tenofovir disoproxil fumarate 300 mg qd** |
| **52** | **male** | **7years** | **726** | **Bictegravir 50 mg + emtricitabine 200 mg + tenofovir alafenamide 25 mg** |
| **59** | **male** | **4years** | **882** | **Bictegravir 50 mg + emtricitabine 200 mg + tenofovir alafenamide 25 mg** |
| **63** | **male** | **5years** | **955** | **Efavirenz 400 mg + lamivudine 300 mg + tenofovir disoproxil fumarate 300 mg qd** |
| **72** | **male** | **10years** | **664** | **Lamivudine + dolutegravir qd** |
| **73** | **male** | **4years** | **327** | **Biktarvy qd** |
| **84** | **male** | **5years** | **563** | **Dolutegravir 50 mg + lamivudine 300 mg qd** |
| **92** | **male** | **10years** | **689** | **Bictegravir 50 mg + emtricitabine 200 mg + tenofovir alafenamide 25 mg** |
| **95** | **male** | **7years** | **340** | **Bictegravir 50 mg + emtricitabine 200 mg + tenofovir alafenamide 25 mg** |
| **97** | **male** | **1years** | **550** | **Bictegravir 50 mg + emtricitabine 200 mg + tenofovir alafenamide 25 mg** |
| **104** | **male** | **1years** | **866** | **Bictegravir 50 mg + emtricitabine 200 mg + tenofovir alafenamide 25 mg** |
| **111** | **male** | **8years** | **1015** | **Bictegravir 50 mg + emtricitabine 200 mg + tenofovir alafenamide 25 mg** |
| **130** | **male** | **2years** | **819** | **Efavirenz 400 mg + lamivudine 300 mg + tenofovir disoproxil fumarate 300 mg qd** |
| **131** | **male** | **2years** | **714** | **Efavirenz 400 mg + lamivudine 300 mg + tenofovir disoproxil fumarate 300 mg qd** |
| **137** | **male** | **5years** | **546** | **Efavirenz 400 mg + lamivudine 300 mg + tenofovir disoproxil fumarate 300 mg qd** |
| **144** | **male** | **5years** | **405** | **Lamivudine + dolutegravir qd** |
| **145** | **male** | **6years** | **601** | **Biktarvy qd** |
| **154** | **male** | **5years** | **977** | **Efavirenz 400 mg + lamivudine 300 mg + tenofovir disoproxil fumarate 300 mg qd** |
| **159** | **male** | **6years** | **505** | **Biktarvy qd** |
| **162** | **male** | **2month** | **361** | **Biktarvy qd** |

**Table S2. Software and packages used in this study**

| **Software / Package** | **Version** | **Purpose in this study** |
| --- | --- | --- |
| FastQC | 0.12.1 | Raw read quality assessment |
| fastp | 0.23.2 | Adapter trimming and read filtering |
| HISAT2 | 2.2.1 | Alignment to the GRCh38 reference genome |
| samtools | 1.9 | SAM-to-BAM conversion and BAM sorting |
| featureCounts | 2.0.1 | Gene-level read counting |
| R | 4.3.3 | Statistical analysis and visualization |
| DESeq2 | 1.42.1 | Differential expression analysis and variance-stabilizing transformation |
| org.Hs.eg.db | 3.18.0 | Gene ID annotation and conversion |
| clusterProfiler | 4.10.1 | GO, KEGG, and GSEA analyses |
| enrichplot | 1.22.0 | GSEA visualization |
| ggplot2 | 4.0.0 | PCA, volcano plots, bar plots, and violin plots |
| ggrepel | 0.9.5 | Non-overlapping text labels |
| dplyr | 1.1.4 | Data manipulation |
| tidyr | 1.3.1 | Data reshaping |
| pheatmap | 1.0.12 | Heatmap visualization |
| VennDiagram | 1.7.3 | Venn diagram plotting |
| RColorBrewer | 1.1.3 | Color palette generation |
| ggforce | 0.4.2 | Ellipse annotation in PCA |
| gridExtra | 2.3 | Multi-panel figure arrangement |
| Cytoscape | 3.10.4 | PPI network visualization |
| cytoHubba | 0.1 | Hub-gene ranking by Degree |
| clusterMaker2 | 2.3.4 | Module detection using MCODE |
